# Apremilast in Refractory Behçet’s Syndrome: A Multicenter Observational Study

**DOI:** 10.3389/fimmu.2020.626792

**Published:** 2021-02-04

**Authors:** Matheus Vieira, Solène Buffier, Mathieu Vautier, Alexandre Le Joncour, Yvan Jamilloux, Mathieu Gerfaud-Valentin, Laurence Bouillet, Estibaliz Lazaro, Stéphane Barete, Laurent Misery, Delphine Gobert, Tiphaine Goulenok, Olivier Fain, Karim Sacre, Pascal Sève, Patrice Cacoub, Cloé Comarmond, David Saadoun

**Affiliations:** ^1^ Sorbonne Universités AP-HP, Groupe Hospitalier Pitié-Salpêtrière, Département de Médecine Interne et Immunologie Clinique, Paris, France; ^2^ Centre National de Références Maladies Autoimmunes Systémiques Rares, Centre National de Références Maladies Autoinflammatoires et Amylose Inflammatoire, Groupe Hospitalier Pitié-Salpêtrière, AP-HP, Paris, France; ^3^ Inflammation-Immunopathology-Biotherapy Department (DMU 3iD), Groupe Hospitalier Pitié-Salpêtrière, AP-HP, Paris, France; ^4^ INSERM 959, Groupe Hospitalier Pitié-Salpêtrière, AP-HP, Paris, France; ^5^ Department of Internal Medicine, Hopital de la Croix-Rousse, Hospices Civils de Lyon, Lyon, France; ^6^ Service de Médecine Interne, CHUGA, Unité Inserm 1036, Université Grenoble Alpes (UGA), Grenoble, France; ^7^ Department of Internal Medicine, Centre de référence national des angioedèmes (CREAK), Grenoble, France; ^8^ Department of Internal Medicine, Haut-Lévêque Hospital, Pessac, France; ^9^ Département Hospitalo-Universitaire Inflammation-Immunopathologie-Biotherapie (DHU i2B), Sorbonne Universités, UPMC Université Paris 06, UMR 7211, Paris, France; ^10^ INSERM, UMR_S 959, Paris, France; ^11^ UF de Dermatologie, Groupe Hospitalier Pitié-Salpêtrière, APHP, Paris, France; ^12^ Department of Dermatology, Brest University Hospital, Brest, France Univ Brest, Brest, France; ^13^ Service de Médecine Interne et Inflammation-Immunopathology-Biotherapy Department (DMU 3iD), Faculté de Médecine Sorbonne Université, Hôpital Saint Antoine, Sorbonne Universités AP-HP, Paris, France; ^14^ Département de Médecine Interne, Hôpital Bichat, Assistance Publique Hôpitaux de Paris (APHP), Institut national de la santé et de la recherche médicale (INSERM) U1149, Université de Paris, Paris, France

**Keywords:** Behçet, apremilast, efficacy, safety, joint, skin, cohort

## Abstract

**Objective:**

Mucocutaneous and joint disorders are the most common manifestations in Behçet’s syndrome (BS) and are frequently clustered in the so-called minor forms of BS. There remains a need for safe and effective treatment for joint lesions in BS. We report the long-term safety and effectiveness of apremilast in refractory joint and mucocutaneous manifestations of BS.

**Methods:**

French nationwide multicenter study including 50 BS patients with either active joint and/or mucocutaneous manifestations resistant to colchicine and/or DMARDs. Patients received apremilast 30 mg twice a day. Primary effectiveness endpoint was the proportion of patients with complete response (CR) of articular symptoms at month 6 (M6), defined as resolution of inflammatory arthralgia and arthritis, with joint count equal to zero.

**Results:**

At inclusion, the median tender and swollen joint count was of 4 [2-6] and 2 [1-2], respectively. The proportion of CR in joint disease at M6 was 65% (n = 15/23), and 17% (n = 4/23) were partial responders. CR of oral and genital ulcers, and pseudofolliculitis at M6 was 73% (n = 24/33), 94% (n = 16/17) and 71% (n = 10/14), respectively. The overall response at M6 was 74% for the entire cohort and 70% for the mucocutaneous-articular cluster (n = 27). The median Behçet’s syndrome activity score significantly decreased during study period [50 (40–60) *vs.* 20 (0–40); *p <*0.0001]. After a median follow-up of 11 [6-13] months, 27 (54%) patients were still on apremilast. Reasons for apremilast withdrawal included adverse events (n = 15, 30%) and treatment failure (n = 8, 16%). Thirty-three (66%) patients experienced adverse events, mostly diarrhea (n = 19, 38%), nausea (n = 17, 34%) and headache (n = 16, 32%).

**Conclusion:**

Apremilast seems effective in BS-related articular disease refractory to colchicine and DMARDs. Discontinuation rates were significantly higher than that reported in clinical trials.

## Introduction

Behçet’s syndrome (BS) is a chronic, relapsing, inflammatory disease of unknown etiology, typically characterized by oral and genital ulcers with several potential systemic manifestations (1). Mucosa, skin, and joint involvement are among the most frequently reported manifestations. These symptoms frequently cluster in the so-called minor forms of BS (2, 3). Mucocutaneous manifestations constitute the hallmark of the syndrome, with the most common skin lesions being pseudofolliculitis and erythema nodosum. Joint involvement, mainly arthralgia, involve half of the patients, and may inaugurate BS (4). In contrast to major organ involvement, mucocutaneous and articular manifestations do not have a major impact on mortality (5, 6), but can be extremely disabling. The main therapeutic goal for these patients is to improve quality of life while minimizing side effects. Despite a wide number of topical and immunosuppressive drugs available in this context, their level of evidence remains limited (7), and the recommended therapeutic lines (i.e., colchicine and disease-modifying antirheumatic drugs - DMARDs) do not effectively control all patients (8). Moreover, following a phenotype-based treatment approach in BS, strategies effective against both mucocutaneous and articular manifestations are increasingly desirable (9).

Apremilast is an orally available small-molecule that selectively inhibits phosphodiesterase 4 (PDE4), and ultimately modulates both anti- and pro-inflammatory downstream mediators. By increasing intracellular levels of cyclic adenosine monophosphate (cAMP), apremilast upregulates interleukin-10 (IL-10) gene transcription, while inhibiting nuclear factor-κB (NF-κB)-driven genes, such as tumor necrosis factor (TNF) (10). Its efficacy has been proven in BS oral ulcers in phases II and III randomized placebo-controlled clinical trials (11, 12), leading to its approval by the FDA in 2019 (13). This effect was further confirmed in short-term small case series (14–16). Nevertheless, the efficacy of apremilast on other manifestations, and specifically on the joints, is still lacking. In addition, the prevalence and impact of its side effects in large real-life cohort with long-term follow-up period has not been assessed.

The present study aims to further investigate the effectiveness and safety of apremilast in a nationwide multicenter cohort of BS patients with refractory joint and mucocutaneous manifestations.

## Patients and Methods

### Patients

We conducted a nationwide observational cohort study within the French Behçet’s network. All patients were adults meeting the criteria of International Study Group for Behçet’s Disease (1), and had either recurrent active joint and/or mucocutaneous manifestations that were refractory to colchicine, conventional synthetic (csDMARDs), and/or biological disease-modifying antirheumatic drugs (bDMARDs). The study was conducted in compliance with the Declaration of Helsinki, and no formal consent from participants was required according to local ethics committees. All data were collected from electronic medical records, including demographic features, BS characteristics at diagnosis, and previous treatments. Data on medications, safety, and disease activity, such as oral and genital ulcers, cutaneous, and articular disease or any other BS manifestations were collected at the time of apremilast initiation, at months 3 and 6 (M3, M6), and at last visit (end of follow-up).

### Design

Apremilast was administered orally by increasing the doses gradually over 1 week up to a dose of 30 mg twice daily. Colchicine, prednisone and other immunosuppressive therapies were allowed if given at a stable dose over the month prior inclusion and during the study period. Patients who needed temporary increase in prednisone dose or any additional immunomodulatory therapy during the study period were considered as non-responders to apremilast.

### Study Endpoints

The primary effectiveness endpoint was the proportion of patients with complete response of joint involvement at M6, defined as resolution of inflammatory arthralgia/arthritis and tender/swollen joint count (TJC, SJC) = 0. Secondary endpoints included (i) the proportion of patients with a complete response of ulcerations (defined as no oral and genital ulcers) (ii) the proportion of patients with a partial response (defined as patients who had a reduction of 50% or more in the number and frequency of oral and genital ulcers, inflammatory arthralgia, arthritis, and joint counts, and skin lesions); (iii) proportion of non-responders (defined as treatment failure and/or the needed for temporary increase in prednisone dose or any additional immunomodulatory therapy during the study period); (iv) effectiveness on other BS manifestations (i.e., ocular, vascular, neurological or gastrointestinal tract involvement); (v) the overall response at M6 for the whole cohort and for the mucocutaneous-articular phenotype (those interrupting treatment before it, regardless of the reason, were considered as non-responders); (vi) BSAS score (17) between baseline and the end of follow up (EOF); (vii) relapse rate under apremilast; (viii) steroids sparing effect of apremilast between day 0 and EOF, and (ix) safety, as all adverse events were prospectively collected during the follow-up.

### Statistics

Data are presented as the median and interquartile range [IQR] for continuous variables and as number (n) and percentage (%) for qualitative variables. Wilcoxon signed rank test with continuity correction was used to compare paired continuous variables. *P* values less than 0.05 were considered significant. Statistical analyses were performed using the software R version 3.6.3.

## Results

### Characteristics of BS Patients

We included 50 patients [27 (54%) females, with median age of 42 (34–48) years]. Main baseline and treatment characteristics are summarized in **Tables 1** and **2**. All patients had active joint and/or mucocutaneous manifestations resistant to colchicine and/or DMARDs. The most common previous manifestations of BS were oral ulcers (98%), arthralgia (76%), genital ulcers and pseudofolliculitis (70%), vascular (22%), and ocular involvement (18%).

**Table 1:** Clinical and demographic characteristics of 50 patients with Behçet’s syndrome.

**Demographic features**	
Age, median [IQR] years	42 [34–48]
Female sex, n (%)	27 (54)
HLA-B51, n (%)^†^	9 (39)
Disease duration, median [IQR] years	5 [1–9]
**Clinical features at diagnosis**	
Oral ulcers, n (%)	49 (98)
Genital ulcers, n (%)	35 (70)
Pseudofolliculitis, n (%)	35 (70)
Erythema nodosum, n (%)	10 (20)
Positive pathergy test, n (%)	4 (8)
Arthralgia, n (%)	38 (76)
Arthritis, n (%)	13 (26)
Vascular involvement, n (%)	11 (22)
Ocular involvement, n (%)	9 (18)
Gastrointestinal involvement, n (%)	4 (8)
CNS involvement, n (%)	3 (6)
**Disease status at the beginning of Apremilast**	
Oral ulcers, n (%)	47 (94)
- Number of lesions, median [IQR]	2 [2–3]
Genital ulcers, n (%)	23 (46)
- Number of lesions, median [IQR]	1 [1-1]
Pseudo folliculitis, n (%)	18 (36)
Erythema nodosum, n (%)	5 (10)
Joint involvement, n (%)	30 (60)
- Arthritis, n (%)	7 (14)
Eye involvement, n (%)	1 (2)
BSAS, median [IQR]	50 [40-60]

^†^HLA-B51 had been performed in 23 patients.

BSAS, Behçet’s syndrome Activity Score; IQR, interquartile range.

**Table 2:** Treatments received as part of Behçet’s syndrome (BS) before and during apremilast.

**Previous treatments during disease course**	
Number of treatment lines, median [IQR]	2 [1-3]
Colchicine, n (%)	49 (98)
Corticosteroids, n (%)	26 (52)
csDMARDs, n (%)^†^	22 (44)
- Number of csDMARDs, median [IQR]	1 [1-2]
bDMARDs, n (%)^‡^	9 (18)
- Number of bDMARDs, median [IQR]	1 [1-5]
**Medications in use before apremilast start**	
Colchicine, n (%)	29 (58)
- Median dose [IQR], mg	1.5 [1-2]
Prednisone, n (%)	15 (30)
- Median dose [IQR], mg	6 [5-15]
csDMARDs, n (%)	11 (22)
- Methotrexate, n (%)	4 (8)
- Azathioprine, n (%)	3 (6)
- Thalidomide, n (%)	3 (6)
- Dapsone, n (%)	1 (2)
bDMARDs, n (%)	5 (10)
- Ustekinumab, n (%)	2 (4)
- Adalimumab, n (%)	1 (2)
- Certolizumab, n (%)	1 (2)
- Secukinumab, n (%)	1 (2)
**Combination treatment with Apremilast **	
Colchicine, n (%)	18 (36)
Prednisone, n (%)	14 (28)
- Median dose [IQR], mg	6 [5–15]
csDMARDs, n (%)	3 (6)
- Methotrexate, n (%)	3 (6)
bDMARDs, n (%)	3 (6)
- Adalimumab, n (%)	1 (2)
- Certolizumab, n (%)	1 (2)
- Ustekinumab, n (%)	1 (2)

^†^Previous csDMARDs included azathioprine, dapsone, hydroxychloroquine, methotrexate, and thalidomide.

^‡^Previous bDMARDs included anakinra, low-dose interleukin-2, secukinumab, tocilizumab, anti-tumor necrosis factor (adalimumab, certolizumab, etanercept, and infliximab), and ustekinumab.

bDMARDs, biologic disease-modifying antirheumatic drugs; csDMARDs, conventional synthetic disease-modifying antirheumatic drugs; IQR, interquartile range.

Ninety-eight percent of patients had already received colchicine, and 52% and 62% had been previously treated with steroids or DMARDs, respectively. Before apremilast treatment, BS patients had received a median number of previous treatment lines of 2 [1-3].

At inclusion, 30 patients (60%) had refractory joint manifestations with a median TJC and SJC of 4 [2-6] and 2 [1-2], respectively. Forty-seven (94%) and 23 (46%) patients had recurrent oral and genital ulcers, respectively. Pseudofolliculitis was present in 18 (36%) patients and erythema nodosum in 5 (10%). Median BSAS was 50 [40-60].

At the time of apremilast initiation, 18 (36%) and 14 (28%) patients continued to receive stable dose of colchicine, and prednisone (median dose = 6 [5-15] mg), respectively. Three (6%) patients pursued csDMARDs (i.e., methotrexate), and three (6%) continued bDMARDs (i.e., adalimumab, certolizumab, and ustekinumab).

### Effectiveness

Six months after apremilast initiation, 65% of patients (n = 15/23) presented complete response (CR) of joint involvement and 17% (n = 4/23) had partial response (PR), while 17% (n = 4/23) were non-responders (**Table 3**). Median TJC and SJC remained zero from M3 until the EOF. Among 22 patients at the EOF with joint involvement, 12 (59%) were complete responders, two (9%) partial responders and eight (36%) had no response.

**Table 3:** Effectiveness of apremilast on articular manifestations.

	Baseline	M3	M6	EOF
n	30	27	23	22
Complete response, n (%)	–	17 (63)	15 (65)	12 (55)
Partial response, n (%)	–	3 (11)	4 (17)	2 (9)
TJC, median [IQR]	4 [2–6]	0 [0–3]	0 [0–2]	0 [0–3]
SJC, median [IQR]	2 [1–2]	0 [0–0]	0 [0–0]	0 [0–0]

EOF, end of follow-up; IQR, interquartile range; SJC, swollen joint count; TJC, tender joint count.

Mucocutaneous response is shown in **Table 4**. The proportion of complete responders for oral and genital ulcers at M6 was 73% (n = 24/33) and 94% (n = 16/17), respectively. At the EOF, no response was seen in 25% (n = 7/28) of oral ulcers and 20% (n = 3/15) of genital ulcers. As for pseudofolliculitis, 71% (n = 10/14) were complete responders at M6, and remarkably no patient had non-response during follow-up. For the two patients with erythema nodosum, one had CR and the other PR at M6. Noteworthy, the only patient presenting ocular involvement at baseline experienced a complete resolution of his refractory keratitis.

**Table 4:** Effectiveness of apremilast on mucocutaneous manifestations.

	M3	M6	EOF
**Oral ulcers**			
n	41	33	28
Complete response, n (%)	26 (63)	24 (73)	15 (54)
Partial response, n (%)	12 (29)	8 (24)	6 (21)
**Genital ulcers**			
n	20	17	15
Complete response, n (%)	15 (75)	16 (94)	11 (73)
Partial response, n (%)	3 (15)	1 (6)	1 (7)
**Pseudofolliculitis**			
n	16	14	11
Complete response, n (%)	11 (69)	10 (71)	7 (64)
Partial response, n (%)	4 (31)	4 (29)	4 (36)
**Erythema nodosum**			
n	4	2	2
Complete response, n (%)	3 (75)	1 (50)	1 (50)
Partial response, n (%)	0	1 (50)	0

EOF, end of follow-up.

The overall response for the whole cohort at M6 was 74% (CR = 48%, PR = 26%). Regarding specifically the mucocutaneous-articular cluster (n = 27), the overall response at M6 was 70% (CR = 30%, PR = 40%). Median BSAS significantly decreased from baseline to EOF (50 [40-60] *vs.* 20 [0-40]; *p* < 0.0001). Among BS patients on steroids, median daily dose of prednisone significantly decreased from baseline to EOF (6 [5-15] *vs.* 5 [5-9] mg; *p* = 0.021). Two (14%) patients discontinued corticosteroids.

A total of 14 patients (28%) experienced BS relapses while on apremilast. Six of them had isolated mucocutaneous reactivations, five presented articular and mucocutaneous concomitant flares, and three experienced exclusively articular activity. Median time to relapse was 6 [4-11] months. A patient who had been presenting complete mucocutaneous response until then developed an unprecedented ileitis after the sixth month of treatment. No other major organ involvement was observed during the study period.

### Safety

Apremilast was discontinued in 23 patients (46%). Treatment interruption was mainly due to side effects (n=15, 30%), and treatment failure (n=8, 16%) (six relapses, one lack of response, and one disease progression). Six patients (12%) presented an early intolerance, with median time to treatment interruption of 7 [5-9] days. Among all AEs requiring discontinuation, gastrointestinal disorders, headache, and sleep disorder were the most frequent, reported in 10 (67%), nine (60%), and three (20%) patients, respectively.

Apremilast dose reduction was tried in seven (14%) patients presenting poor tolerance to conventional dosage despite good initial response. After a median follow-up of 11 [6-13] months for the entire cohort, 27 (54%) patients were still on apremilast.

Thirty-three (66%) patients experienced adverse events (AE), with median time to onset of 4 [1-4] weeks. Most common side effect included diarrhea (n = 19, 38%), followed by nausea (n = 17, 34%) and headache (n = 16, 32%). Adverse events frequency is detailed in **Table 5**. Two (4%) patients experienced suicidal ideation leading to treatment discontinuation, with one of them being hospitalized for its management. Moreover, four (8%) patients experienced infections, namely mycobacteria reactivation, cat scratch disease, herpes simplex, and an acute gastroenteritis. None of them were on concomitant DMARDs.

**Table 5:** Adverse events during apremilast treatment.

≥ 1 Adverse events, n (%)	33 (66)
Number of adverse events, median [IQR]	2 [1–3]
Time to onset, median [IQR] weeks	4 [1–4]
Adverse events leading to discontinuation, n (%)	15 (30)
Adverse events leading to hospitalization, n (%)	1 (2)
**Adverse events frequency**	
Diarrhea, n (%)	19 (38)
Nausea, n (%)	17 (34)
Headache, n (%)	16 (32)
Abdominal pain, n (%)	10 (20)
Sleep disorder, n (%)	9 (18)
Fatigue, n (%)	5 (10)
Infection, n (%)	4 (8)
Suicidal ideation, n (%)	2 (4)
Depression, n (%)	2 (4)
Anorexia, n (%)	2 (4)

IQR, interquartile range.

## Discussion

This multicentric study reports the largest real-life cohort of patients with BS treated with apremilast. The main conclusions drawn are: 1) 65% of BS patients with refractory joint manifestations at 6 months had a complete response; 2) Discontinuation rates were three times higher than that reported in clinical trials; and 3) BS patients with refractory skin disease respond to apremilast.

Management of mucocutaneous and articular symptoms in BS can be challenging. Current recommendations place colchicine as the first-line option, followed by several DMARDs, such as azathioprine, thalidomide, interferon-alpha and tumor necrosis factor inhibitors for refractory cases (8, 18). While some of these drugs have conflicting results in terms of efficacy, others have safety concerns, making management even more difficult (7). With the increasing availability of bDMARDs, new targets have been assessed in BS recently. Ustekinumab – a monoclonal antibody targeting interleukin-12 and -23 – was evaluated in a prospective, open-label study, showing promising results in mucocutaneous and articular manifestations resistant to colchicine in BS (19). In a retrospective study, the anti-interleukin-17 secukinumab was evaluated in the mucocutaneous-articular cluster refractory to initial treatment, revealing itself as a potential alternative in this subgroup (20). Herein, we report encouraging data on apremilast for BS refractory mucocutaneous-articular phenotype, notably regarding joint disease. The proportion of patients experiencing articular improvement at M6 was up to 82%, with 65% of complete responders. After the first 6 months of treatment, 64% of BS patients were still being improved. So far, only one small study reported articular outcomes in 14 BS being treated with apremilast. A complete response was obtained in 28% of cases over a 3-month period (16). In contrast, apremilast’s efficacy has been better described in psoriatic arthritis (PsA). Similar to our results, a real-life PsA cohort showed that 61% of patients were responders at 6 months (21). Another real-life study with 131 PsA patients highlighted 40% of remission or low disease activity at 3 months and a drug-retention rate of 72% at 6 months (22). In a pooled analysis from clinical trials using the American College of Rheumatology (ACR) response criteria, 55% and 26% of PsA patients receiving apremilast maintained an ACR20 and ACR50 response at 1 year, respectively (23). In clinical trials, 72% of PsA patients were still on apremilast after a year. Despite its superiority against placebo, apremilast is reported to have low to moderate efficacy when compared to other bDMARDs in active PsA (24). The greater efficiency highlighted in our study may lie in the fact that BS presents with milder articular features (e.g., absence of bone erosions, arthralgia rather than arthritis).

In the absence of phase IV studies, the long-term safety of apremilast is unknown in BS. In BS clinical trials, most patients (71%–91%) experienced at least one adverse event (11, 12). Along this line, we found a similar frequency of AE (66%). Despite this high rate, AEs leading to discontinuation in BS controlled studies did not exceed 11% (11, 12). Strikingly, 30% of our patients interrupted apremilast owing to poor tolerance, of which 12% discontinuation as of one week. Another 16% ceased treatment due to failure, which is also higher than the 2%–7% seen in phase II/III placebo-controlled studies (11, 12). Indeed, a gap between clinical trials and real-life studies has been noted in other apremilast label indications. In PsA, pooled data from phase III trials reported withdrawal due to AE in 7.6% of patients over a 1-year period (25). Conversely, real-life studies have demonstrated higher rate of apremilast discontinuation ranging from 20% to 38% (21, 26). This contrast seems less pronounced in psoriasis, as 3-year pooled trial data showed 11% of AE resulting in discontinuation (27), whereas in real-life cohorts this rate varied between 16% and 19% (28, 29). Interestingly, a network meta-analysis evaluating safety among 12 different bDMARDs in PsA pointed out apremilast as the only medication with significantly higher chance of withdrawal due to AE (30).

Regarding the type of AE, a similar profile was reported in BS, PsA or psoriasis studies, with diarrhea, nausea, and headache accounting for the most common events (11, 12, 14, 15, 25, 27). Although gastrointestinal side effects represented the leading symptom motivating discontinuation in our cohort, two patients (4%) interrupted apremilast because of suicidal ideation. This serious AE has been consistently reported in post-marketing surveillance and continued pharmacovigilance is warranted (31). Upper respiratory tract infection has been the most reported infection in association to apremilast. Although we did not find any cases of it, 8% presented infections in our study, notably one mycobacterial reactivation. So far, no case of mycobacterial infection has been reported under apremilast in BS. In a large database cohort evaluating immunosuppressants infectious risk among psoriasis and PsA patients, only two tuberculosis codes were identified concomitantly to apremilast prescription over a follow-up of 12,842 person-years (32).

Our study highlighted apremilast’s effectiveness in BS refractory skin disease. Patients with pseudofolliculitis achieved a sustained a complete response in nearly 70% over the study period. Phase III placebo-controlled study did not show efficacy of apremilast in BS skin disease (12), and two case series reported contrasting responses (100% vs. 0%) (15, 16). In psoriasis skin lesions, the long-term benefit of apremilast has been well established. Over a 2-year period, up to 52% of psoriasis patients maintained ≥ 75% reduction in Psoriasis Area and Severity Index (PASI) score from baseline (33). As BS share several common features with psoriasis (34), it is not surprising that apremilast could also work for BS manifestations other than oral ulcers. Finally, we confirm the efficacy of apremilast on BS oral ulcers consistently with phase III trial results (12). When compared to these, we found slightly higher complete response rates at 3 months for oral (63% vs. 53%) and genital ulcers (75% vs 71%). Real-life case series had further confirmed this significant impact on oral ulcers and demonstrated a positive trend on genital ulcers in smaller samples (14–16).

Our study has some limitations. The continuation of systemic therapy (i.e., colchicine, DMARDs) at steady-state doses was possible during apremilast treatment, and as there was no protocol limiting its concomitant use, this could represent a potential confounder in effectiveness evaluation. Nevertheless, colchicine and DMARDs were already at their optimized dosage, and only two patients needed additional combination therapy during the study, being considered as non-responders. Moreover, compared to the phase III trial where only 50% of patients had been previously received colchicine (12), all of our patients were refractory to colchicine, DMARDs, and/or prednisone.

In conclusion, this nationwide multicenter cohort study shed new lights on the effectiveness and tolerability of apremilast in BS patients with refractory joint and mucocutaneous manifestations. Besides oral ulcerations, apremilast seems to improve refractory joint and skin manifestations in those who manage to persist on treatment. However, the discontinuation rate was high mainly for safety issues. This raises the question of whether this treatment can be used for long-term management of BS.

## Data Availability Statement

The raw data supporting the conclusions of this article will be made available by the authors upon reasonable request.

## Ethics Statement

Ethical review and approval was not required for the study on human participants in accordance with the local legislation and institutional requirements. The ethics committee waived the requirement of written informed consent for participation.

## Author Contributions

MVi, MVi, CC, and DS conceptualized this work. MVa, SuB, MVa, AL, YJ, MG-V, LB, EL, SaB, LM, DG, TG, OF, KS, and PS were involved in data collection and analysis. MVi wrote the initial draft of the manuscript. Authors contributed to the article and approved the submitted version.

## Conflict of Interest

DS reports grants and personal fees from AMGEN, during the conduct of the study, grants and personal fees from ABBVIE, grants and personal fees from ROCHE-CHUGAI, grants and personal fees from SANOFI, and grants from GSK, outside the submitted work.

The remaining authors declare that the research was conducted in the absence of any commercial or financial relationships that could be construed as a potential conflict of interest.
